# ‘It’s Important to Make Changes.’ Insights about Motivators and Enablers of Healthy Lifestyle Modification from Young Aboriginal Men in Western Australia

**DOI:** 10.3390/ijerph16061063

**Published:** 2019-03-24

**Authors:** Kimberley H. Seear, Matthew P. Lelievre, David N. Atkinson, Julia V. Marley

**Affiliations:** 1 The Rural Clinical School of Western Australia, The University of Western Australia, Broome WA 6725, Australia; david.atkinson@rcswa.edu.au (D.N.A.); julia.marley@rcswa.edu.au (J.V.M.); 2 Derby Aboriginal Health Service, Derby WA 6728, Australia; matthew.lelievre@nwrh.com.au; 3 Kimberley Aboriginal Medical Services, Broome WA 6725, Australia

**Keywords:** indigenous, type 2 diabetes, prevention, motivation, change, healthy lifestyle, health education, health behaviour, social determinants of health, qualitative research

## Abstract

Lifestyle modification can improve the health of people with or at risk of non-communicable diseases; however, initiating and maintaining positive health behaviours including healthy eating and physical activity is challenging. Young remote Aboriginal people who had successfully made significant healthy lifestyle changes were sought out to explore how they achieved this success. Four Aboriginal men aged 20–35 years were identified and consented to participate. Their perceptions of motivation for change, strategies, and facilitators and barriers were explored through in-depth interviews. Themes developed from the interviews included self-efficacy, self-reliance, and increased knowledge and altered health beliefs underpinning change. Participants with diabetes were highly motivated to avoid diabetes complications and had a strong belief that their actions could achieve this. In a setting with high levels of disadvantage, participants had relatively favourable socioeconomic circumstances with solid social supports. These findings highlight that lifestyle modification programs that foster internal motivation, enhance key health knowledge, and modify health beliefs and risk perception are needed. Increasing diabetes awareness among at-risk young people is important, emphasising the largely preventable and potentially reversible nature of the condition. Broad health improvements and individual changes will be facilitated by equitable socioeconomic circumstances and environments that support health.

## 1. Introduction

Physical activity and an appropriate diet are key factors in good health and chronic disease prevention but are commonly lacking in modern lifestyles, often requiring considerable effort in obesity-promoting environments [1,2]. Large-scale trials such as the US Diabetes Prevention Program have demonstrated that lifestyle modification can be achieved and sustained over several years [3], and can prevent type 2 diabetes in people at high risk [4]. These findings have been applied in various real-world studies, with some success [5].

The mean age of participants in published reports of lifestyle intervention programs, including with Indigenous participants in a range of Western countries, is commonly above 40 years [5,6,7,8]. However, changes leading to diabetes commence much earlier and overt type 2 diabetes is now increasingly found in younger people. This is more common in populations who have been disadvantaged, including Indigenous peoples who are impacted by historical or transgenerational trauma and socioeconomic inequalities [9]. In Australia, Aboriginal and Torres Strait Islander people have higher rates of type 2 diabetes than non-Indigenous Australians in all age groups, and particularly in younger age groups. For ages 10–39, the incidence was reported as 126.7 per 100,000 population, compared with 35.7 per 100,000 in non-Indigenous Australians in 2006–2011 [10].

While health-promoting changes to social and physical environments are needed, there should also be immediate support for positive health behaviours in individuals, including healthy eating and physical activity [11]. Efforts to support individuals and groups may be enhanced by drawing on the successful experiences of others. For example in young-onset type 2 diabetes management, the utilisation of peers who have made lifestyle changes has been recommended to support others and also enhance knowledge about predictors of successful and sustained change [12]. However, there is limited published research about how successful lifestyle modification can be achieved by young Indigenous people.

Lifestyle modification is a cornerstone of type 2 diabetes prevention as well as its management. The aim of this study was to identify how and why some Aboriginal people in a remote Australian town have made lifestyle changes that improved their health and to use these insights to assist others, including by informing the development of a localised diabetes prevention program.

## 2. Materials and Methods 

### 2.1. Ethics Approval and Consent to Participate

This study was part of a diabetes prevention project endorsed by the Board of Derby Aboriginal Health Service. It was approved by the Western Australian Aboriginal Health Ethics Committee (reference 724) and supported by the Kimberley Aboriginal Health Planning Forum Research Subcommittee. Participants provided written informed consent to participate, and for publication of research data gathered for this study. Two participants preferred their real name to be used and provided additional written consent for this.

### 2.2. Setting and Participants

Participants were Aboriginal people living in Derby, a remote town in North West Australia with an estimated population of around 3300, of whom approximately half are Aboriginal [13]. Derby’s socioeconomic profile is among the most disadvantaged in Australia [14]. Derby and the wider Kimberley region has a range of strong Aboriginal organisations working for continued health improvements and assertive Aboriginal cultural practices and well-being in their communities. Intended interviewees were patients of Derby Aboriginal Health Service (DAHS), younger than 40 years and with or at risk of type 2 diabetes, who had made lifestyle changes that had led to sustained positive changes in risk for diabetes or diabetes control. Suitable participants were sought between September 2016 and January 2017. All doctors at DAHS were contacted by the DAHS research liaison officer. The interviewer also made a presentation to doctors, nurses and other health workers at a staff meeting and distributed recruitment flyers to staff within the organisation. 

Despite the above efforts, only four people meeting the inclusion criteria were identified; all agreed to participate. Although men and women were sought, all identified participants were men. Two patients were informed about the study by their regular doctor at DAHS, who had noted their improved glycaemic control in conjunction with lifestyle changes and weight loss. A further two DAHS patients volunteered to be interviewed, after hearing about the study through DAHS networks.

### 2.3. Interviews 

The interviewer was a female non-Indigenous graduate research student living in Derby. Individual interviews were at a location decided by the participant, most commonly the researcher’s office, and lasted 30 to 60 minutes. An interview guide was developed in conjunction with DAHS staff members and used flexibly in a context of ‘yarning’. Characterised by informal and interactive conversation, yarning is considered a culturally appropriate way to provide and seek information. Bessarab and Ng’andu [15] demonstrated its value as an Indigenous research method, including for semi-structured interviews centred on participants’ stories. Consistent with a phenomenological research approach (where sample sizes of 3–10 are common) [16], topics included the changes participants made and why, what made changes easier or harder, how others reacted, and advice or ideas for helping others to make changes. Broad questions are included in the Appendix A. All participants were very forthcoming in sharing their stories, with a view to helping others. Participants were given a $25 store gift card to thank them for their time.

### 2.4. Data Analysis

Interviews were digitally recorded and transcribed verbatim by the interviewer. The interviewer then descriptively coded the data (using NVivo for Mac Version 11, QSR International, Melbourne, Australia). The interview guide topics provided a broad framework; additional and more specific codes evolved as the transcripts were organised by topics in the participants’ narratives. An Aboriginal male allied health professional, with previous research experience, independently reviewed all data. The two research team members then met to discuss their interpretations of the data. No changes to the codes were required at this time. Data were analysed thematically by the interviewer and health professional with reference to the objective of the research and the participants’ unique stories, noting commonalities and differences [17], and illustrative quotes were jointly chosen. Following this analysis, participants were given the option to review identified themes and the associated data they had provided; three of four took part in this respondent validation. None requested changes to the data they had provided, and all accepted the nominated themes. 

## 3. Results

The four participants were residents of Derby, male, employed, and aged between 20 and 35 years. During recruitment, several health care staff had stated it was uncommon to encounter patients who made marked healthy lifestyle changes. Three of the men had been diagnosed with diabetes when aged in their twenties. The fourth did not have diabetes, but had made lifestyle changes leading to substantial weight loss (>30 kg). Two participants had relocated to Derby earlier in the year, and two had lived in Derby for most of their life. Two participants requested that their real name be used in this article. A pseudonym is used for the other participants, indicated by an asterisk with first use.

There were five prominent themes in participants’ stories.

### 3.1. Potential Diabetes Complications as Motivator for Change

Participants with diabetes made lifestyle changes in response to their diagnosis. However, they felt understanding of the disease was key. Motivation for lifestyle changes was largely attributed to risk of serious diabetes complications.

‘I don’t want the complications when I’m old you can go blind, I didn’t know that.’

(Jason*)

‘And then it can lead to dialysis and I didn’t want that, and heart problems and things, so it was scary.’

(Marcus*)

‘Earlier in the year the doctor said to me, and the diabetes educator "you look like you’re almost ready for dialysis”. I don’t think so, I’m 33 years of age, and I swore and I said, “I’m not going to be sitting on that effing machine for 4 hours out of my day for 3 days a week, I don’t think so.”’

(Laurence)

Further, participants spoke candidly about their motivation to avoid dying prematurely due to diabetes.

‘I think that willingness to live and watch my kids grow up, that was, that’s it, you know.’

(Marcus)

‘The thought of dying early gives you a bit of energy, ’cos diabetes—you don’t want to really mess with that I know I don’t want to die early, I want to live a long and healthy life.’

(Jason)

‘I’m determined to live longer, I don’t want to be one of those, you know, statistics [of early Aboriginal mortality].’

(Laurence)

### 3.2. Knowledge as a Foundation for Change

Increased knowledge, including about the influence of lifestyle behaviours on health, empowered participants to make changes. Health professionals were a source of information, and participants also did their own research using the internet. Prior to this, participants had limited knowledge about diabetes risk factors and the possibility of young onset.

‘I was quite shocked when I found out that I had it. It’s like, ‘I’m so young’, you know, ‘this is not what my life is supposed to be’ Even though on both sides of my family diabetes is hereditary, I didn’t really understand.’

(Jason)

‘My nanna had it and it was in our family, but no-one young had it [you think], you’re young, you don’t get that sort of stuff, it doesn’t happen to you you just worry about the day and just go along and think that you’re going to be fine.’

(Marcus)

All participants spoke of a need for additional healthy lifestyle education for young people, particularly in schools, to facilitate chronic disease prevention.

‘Oh my god I never wanted to be a diabetic, and it’s just not nice knowing like, it’s important to make changes and yeah that other people know before they can get to that stage, you know, to do something about it.’

(Jason)

Some participants also described being physically fit as a teenager, followed by rapid weight gain with changed behaviours.

‘It was weird, funny sort of, how quick it happened, and just when you’re in your own flesh the changes happen so quick. ’Cos I remember when I was in Year 10 I was really fit, playing good football, playing good basketball and then just over two years later I was an extra like 50 kilos heavier and just like really, really lethargic, really unhealthy It will catch up with you and it catches up really quick.’

(Trevor)

‘I wish I had the knowledge when I was younger. Well because at school I was fit, you know, I was healthy, I was not overweight but I didn’t give a shit ’cos I ate whatever I wanted. But if I had known at school, which we didn’t have much of this education, like you know how ATSI [Aboriginal and Torres Strait Islander]—Indigenous—people have a high rate of getting diabetes or whatever, we didn’t have any things like that at school, no-one told us that, no-one If I knew, maybe I could have changed it.’

(Jason)

All participants discussed past unhealthy eating habits without consideration of associated health risks at that time. Participants with diabetes also reported previous heavy weekend alcohol consumption without considering health impact.

‘It’s not that I drink every night, I drink every second weekend, or if I’m in [a larger town], but it’s when I drink I don’t stop.’

(Jason)

‘I didn’t drink every day, like I worked and then come Friday, Saturday it was just a binge you know, and fatty foods and chocolate and all that sort of stuff.’

(Marcus)

‘I think the main cause and contributor for my type 2 diabetes was around that alcohol intake I used to have fun on the weekends, the way I thought of it was that I work damn hard all week, it’s my privilege and right if I want to get drunk on the weekend with friends and go party and whatever else.’

(Laurence)

All reported satisfaction with reducing or ceasing alcohol consumption in response to diabetes diagnosis, which was accompanied by increased health knowledge. However, one participant discussed losing some social relationships where alcohol consumption had been prominent. 

### 3.3. Belief That Changes Would Have a Valued Effect

For participants with diabetes, changes in beliefs and attitudes associated with their diagnosis motivated lifestyle modification. In the participant without diabetes, perceiving a need and possibility for change was associated with a desire to improve and prolong sports participation. He had previously considered his weight to be unchangeable, and the alteration of this belief was essential for changed health behaviour.

‘I felt that that was who I was, that I was just this big kid, there was nothing I could do about it People need to see where these choices will take them if they don’t change but also that you can change and being fat, being big is not, it’s not you, it’s something that you can and you should take steps to change. Because I, for years I identified myself as that, but it wasn’t until I guess now that I realise that it’s not, doesn’t define me, it’s not a part of me, and I’m so much more better for it in a multitude of ways.’

(Trevor)

Among participants with diabetes, a belief that their diabetes status and outcomes could be changed was a vital factor in initiating and persisting with new health behaviours.

‘If you don’t control it, it’s something you’re stuck with the rest of your life. And I’m only young so I know what I need to do to, you know, get rid of it basically.’

(Jason)

‘I think having factual information and evidence-based examples that, yes, diabetes can be overturned and you can go back to living a fully normal life not ‘it’s never going to go away’, you can kill a person’s hope.’

(Laurence)

### 3.4. Self-Reliance, with Social Support

Knowledge contributed to self-determination and internal motivation. Participants exhibited high self-efficacy with a strong sense of control over their health.

‘I know that, in my head, I’m determined to live longer, and nobody else can make me live longer except me.’

(Laurence)

‘At the end of the day, you can have all the support in the world—you have to do it yourself. Only you can make all the changes I think the change begins with yourself, that’s why you should believe in yourself.’

(Jason)

While they exhibited a strong sense of self-reliance, all participants spoke of having supportive family and friends, manifested in various ways:

‘[My wife] said, “Well this will be a good turnaround for everyone”, so it was good, we did it together as a family.’

(Marcus)

‘My household knows that I have diabetes now, so they’re very like, “you can’t drink that” or “you can’t eat that” [the household hasn’t made changes], it’s something I wouldn’t expect them to do, I need to do that myself.’

(Jason)

‘Some of them actually say, “Oh I’ll come train with you”, so it’s like that peer motivation type stuff, and as small as it is, it’s actually quite big because it sort of gives you belief that people actually believe in you.’

(Laurence)

This participant also discussed a disparaging comment from a family member about his slow rate of weight loss, elucidating the potentially damaging effects:

‘It can be quite crushing, so in the end I said, “Look, what you said can actually make a huge impact on whether or not I continue to go through this, and if you don’t have anything nice to say then don’t say it.”’

(Laurence)

### 3.5. The Difficulty of Change

Participants spoke of the difficulty in changing long-term health behaviours, until their persistence with lifestyle modification created new habits. All referred to the importance of trying to establish healthy behaviours from an early age in others.

‘I think both [changing diet and exercise] are hard, unless you’re brought up doing those things from a kid or, you know you were like, yeah we play sport in school but then we get to a thing where you work and then home so it’s hard. But once you get, it’s called like a routine, it just becomes so natural.’

(Marcus)

Participants generally found that making slow, small changes was a useful approach, building on these changes over time.

‘I believe in taking baby steps, you don’t rush into things, if things don’t work out you get disheartened I took advice from my doctor, you know, to take things slow. It works out better in the end. Don’t set up these really extreme goals.’

(Jason)

‘I actually started to start small with exercise and work my way up ’Cos I’ve got bad knees I can’t play sport, so I just started walking.’

(Marcus)

In addition to general difficulties in lifestyle change, one participant (Laurence) mentioned the impact of transgenerational trauma, along with social determinants of health behaviours. He spoke about this impeding the changes that may be required for good physical health:

‘Sometimes people are not ready to change—they’ve got so much else going on in their life. People put mental health before physical health, you know. In the mind/spirit/body stuff, body comes last. Aboriginal health is a lot about social-emotional health; this needs to be nurtured before you can do the physical stuff.’

**Figure 1:**
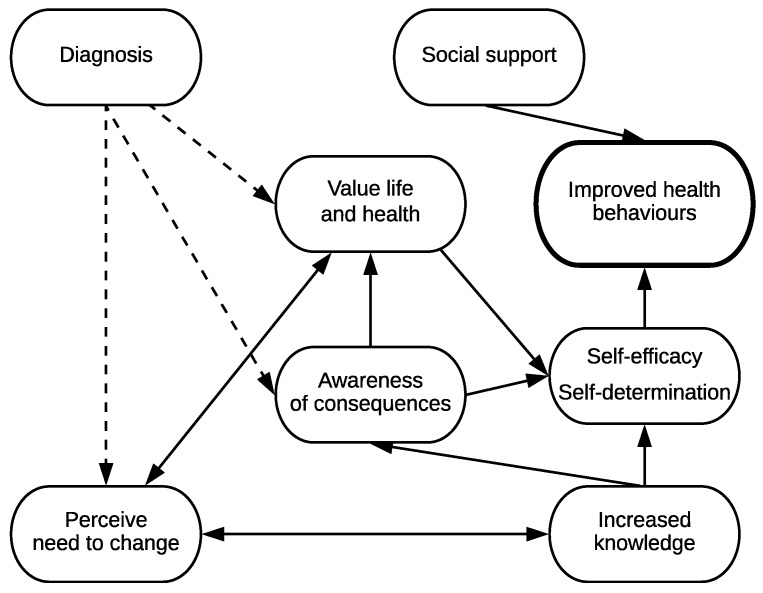
Factors influencing change. Note: Dashed lines reflect that not all participants had diabetes.

The apparent relationships between key factors influencing improved health behaviours in these participants is represented in Figure 1.

## 4. Discussion

This study contributes to understanding successful healthy lifestyle change in the context of a remote town with a large Aboriginal population. Healthy behaviour changes were related to enhanced knowledge and skills, contextual factors, and personal attributes of these men including self-efficacy. Each participant changed their health behaviours after becoming aware that it was needed for what they valued in life, and that it would have beneficial effects. Participants with diabetes were motivated by the prospect of its consequences and a belief that lifestyle changes could prevent these. All participants generally reported a lack of previous knowledge about type 2 diabetes, including risk factors such as family history, physical inactivity, and an unhealthy diet. None had perceived themselves to be susceptible to type 2 diabetes as young people.

The knowledge participants gained, from health professionals and their own research, promoted self-determined healthy behaviours [18]. Detecting benefits of lifestyle changes perpetuated motivation. However, change was difficult, particularly until healthy behaviours became habitual. These findings are consistent with the Health Belief Model theory of health behaviour and behaviour change, which has relevance in various cultures [19]. To engage in preventive health behaviour, the model notes that a person first needs to perceive their susceptibility to, and the seriousness of, a potential consequence. They then need to believe the behaviour will be beneficial, with no significant barriers to engaging in it. A ‘cue to action’ may also be needed [20]. For most participants in this study this was being diagnosed with diabetes, prompting consideration of serious complications (e.g. kidney failure, blindness, amputation) and early death. This was accompanied by a change in perceived risk. 

Self-efficacy was added to the Health Belief Model [21] and has the highest predictive value for healthy behaviour [22]. This is central to motivation, change and persistence [23]. Participant changes typically occurred within a context of social support from family, friends and health professionals. Social relationships are particularly important in Indigenous cultures, possibly making strong positive social supports even more crucial to a favourable outcome in this setting. It is possible that these participants’ high self-efficacy for healthy lifestyle changes reflects more general self-efficacy; however, it is a factor amenable to intervention through social modelling and experiences of success [22]. 

The men in this study made changes without participating in a lifestyle intervention program, but provide a model for desired program outcomes. The willingness of these men to share their experiences and insights to aid others, including to help design potential intervention programs, is greatly valued. Their high level of internal motivation and autonomy is at odds with many lifestyle modification programs that assume a need for periodic, ongoing, external reinforcement [24]. Programs may benefit from promoting autonomous motivation, for sustained lifestyle change [24,25], while recognising that some people will initially need more support to achieve successes that build self-efficacy [23].

While knowledge is obviously not sufficient for lifestyle change, it is fundamental [23]. The men in the current study asserted the need for clear and increased communication of health information, reinforcing findings from a study of Indigenous men of a similar age in three other Australian locations [19]. Participant descriptions of previous unhealthy behaviours with limited health knowledge and awareness are consistent with other research. This includes a qualitative study with overweight and obese participants (predominantly African-American women) aged 18–29 years, with and without pre-diabetes. Unhealthy behaviours were attributed to lack of health knowledge such as guidelines for healthy eating and physical activity, and perceiving behaviours or weight to be insufficiently unhealthy to necessitate change [26].

In addition to necessary practical knowledge and strategies [27], the current study supports the importance of communicating the effects of lifestyle behaviours on health [23], including diabetes risk. As in this study, formative research in First Nations communities in Canada found that participants knew people with diabetes but had little knowledge about the disease [28]. A systematic review including 120 qualitative studies involving people with type 2 diabetes identified that participants could be strongly motivated for dietary change by fear of type 2 diabetes complications [27]. The current study indicates that familiarity with diabetes complications in younger people may be important. Participants also highlighted the benefits of communicating recent research findings that diabetes is potentially reversible [29,30,31].

Heavy drinking was a topic raised by each participant with diabetes. In Australia overall, the dominant social norms supporting alcohol use have important public health implications [32]. As discussed by one participant, this aspect of lifestyle change can especially violate social norms. It should be noted that non-Indigenous Australians are more likely to be alcohol consumers than Indigenous Australians [33]. Of people who do drink alcohol, however, Indigenous Australians have been more likely to report consumption at levels posing high short-term risks [33].

While interviews were centred on participants’ experiences and perspectives, the broader context needs to be considered. The socioeconomic factors of income, employment and education profoundly affect health [33] and our findings are suggestive of their significance for potential healthy lifestyle changes in disadvantaged settings. All participants in this study had good health literacy (including digital health literacy) and employment. This is noteworthy because the unemployment rate in the Shire of Derby/West Kimberley is around 24 per cent [34]. The type of participant group affects the transferability of findings, while also providing additional insight into contexts supporting behaviour change. As one participant elucidated, a person may not be able to address physical health if they do not have sufficient social-emotional health. This is supported by findings from a diabetes and renal disease project in the Western Australian Goldfields, with mainly Aboriginal participants, which identified the influence of stress on unhealthy lifestyle behaviours [35]. Several other studies have identified stress as a barrier to diet modification [27]. We recommend that the social determinants of health and social and emotional well-being be directly investigated in future research regarding lifestyle change, including in relation to self-efficacy.

A major strength of this study is that it is centred on the lived experiences of people who made positive health changes. While descriptions of health-related difficulties experienced by people from minority groups are common, this study instead focused on successful lifestyle changes. The purposive sampling strategy yielded a small number of participants, which may have been reflective of the difficulties facing many people in this setting. Furthermore, not all participants were long-term Derby residents. The sample size was sufficient to fulfil the objective of this study [16,36]; however, wider selection criteria may have enabled identification of others with relevant insights. 

Broader behaviour-related selection criteria may have yielded female participants, who were not intentionally excluded from this study. It has been reported that women with diabetes experience greater challenges with dietary change than men, and may be more likely to prioritise family food preferences instead of their own healthy eating intentions [27]. Further gender-related studies may be useful. It is a potential limitation that the interviewer was not of the same gender and culture as participants. Alternatively, this may have facilitated story-sharing [37]. The involvement of a local, Aboriginal, male allied health professional strengthened the analysis and interpretation of the data. 

## 5. Conclusions

This study highlighted the importance of thorough health knowledge as well as belief about personal capacity to improve health, as part of motivation for lifestyle changes. The men in this study advocated for increased education about healthy lifestyles and the potential consequences of negative health behaviours. This study indicates that an understanding of diabetes is important, including young-onset diabetes, given its prevalence in Aboriginal communities. Healthy behaviours may be motivated by knowledge of diabetes susceptibility, seriousness, and the potential for prevention of both the disease and its consequences. Participants considered it particularly important for healthy lifestyles to be supported from an early age, before unhealthy behaviours are fully established and require strong motivation and persistence to change. In addition to insights about individual lifestyle modification for use in interventions, this study supports the crucial role of social and economic resources in enabling the potential for good health. This includes employment and income, health literacy, and supportive relationships.

## Appendix A

*Interview Questions*

Why did you make changes, how did that come about?

What changes did you make?

What was it like when you made the changes?

Was there anything hard about making the changes?

Was there anything easy about making the changes?

How did other people react about you making the changes?

What would you say to other people who were thinking about making changes?

Is there anything else we haven’t talked about that you think is important?
